# Analysis and Screening of Reproductive Long Non-coding RNAs Through Genome-Wide Analyses of Goat Endometrium During the Pre-attachment Phase

**DOI:** 10.3389/fgene.2020.568017

**Published:** 2020-10-23

**Authors:** Linjun Hong, Qun Hu, Xupeng Zang, Yanshe Xie, Chen Zhou, Xian Zou, Yaokun Li, Ming Deng, Yongqing Guo, Guangbin Liu, Dewu Liu

**Affiliations:** ^1^College of Animal Science, South China Agricultural University, Guangzhou, China; ^2^Lingnan Guangdong Laboratory of Modern Agriculture, Guangzhou, China; ^3^State Key Laboratory of Livestock and Poultry Breeding, Guangdong Key Laboratory of Animal Breeding and Nutrition, Guangdong Public Laboratory of Animal Breeding and Nutrition, Institute of Animal Science, Guangdong Academy of Agricultural Sciences, Guangzhou, China

**Keywords:** RNA-seq, goat, endometrium, lncRNA, implantation

## Abstract

Reproduction in goat is highly impeded by implantation failure. Of concern, the underlying mechanism leading to embryo implantation remains unclear. In this study, deep sequencing was employed through strand-specific Ribo-Zero RNA-Seq to characterize transcriptome changes in the endometrium during the maternal recognition of pregnancy. A total of 996 differential transcripts (115 lncRNAs and 881 mRNAs) existing between the pregnant and non-pregnant endometrium were revealed through bioinformatics analysis. The screening was performed on lncRNAs (XR_001918173.1, LNC_002760, and LNC_000599) and LNC_009053, to determine their potential role in regulating the synthesis of retinol and endometrium remolding through the proteasome pathway, respectively. The hypothesis of whether certain lncRNAs, namely, LNC_007223, LNC_005256, and LNC_010092 could play important roles in embryo implantation was tested. These novel findings are of paramount relevance to further elucidate the molecular mechanisms of embryo implantation and uncover new targets to improve goat reproduction.

## Introduction

Goats are important economic animals as they provide agricultural products, including milk, wool, and meat (Zhan et al., 2016). Goat farming significantly depends on efficient reproduction; however, it is affected by endometrial receptivity, associated with a successful establishment of pregnancy in mammals (Simmons and Kennedy, 2002). Receptive endometrium referred as “window of implantation” is a temporal and spatial phenomenon that necessitates a synchronized and successful molecular dialogue between the conceptus (embryo and associated extra-embryonic membranes) and endometrium (D’Hauterive et al., 2002; Xia et al., 2010). In domesticated ruminants, for instance, a goat, the embryo-maternal communication becomes evident when the elongating conceptuses release a surge of interferon-τ (*IFNT*) on 15–17 days of gestation (Bazer et al., 1991; Spencer et al., 2007b). IFNT is the maternal pregnancy recognition signal that potentially inhibits the expression of estrogen receptor α and mediates antiluteolytic actions (Bazer et al., 1991, 2011). Previous studies revealed that, apart from the antiluteolytic actions, IFNT acts on the endometrium to either improve or induce the expression of IFNT-stimulated genes (ISGs) that are hypothesized to regulate endometrium receptivity to embryo implantation and pregnancy (Hansen et al., 1999; Spencer et al., 2004b). Moreover, the uterine endometrium undergoes dramatic morphological and functional changes induced by pregnancy recognition signal to prepare the endometrium to be receptive for the qualified embryo implantation (Pan et al., 2007; Altmäe et al., 2013). However, impaired uterine receptivity is among the major factors associated with embryo transplantation failure despite the existence of good quality embryos (Macklon et al., 2006; Ai-Guo et al., 2011). Therefore, uncovering the associated molecular regulation mechanisms of endometrial changes during the period of “window of implantation” will be of paramount importance in achieving successful implantation.

Previous studies revealed several genes involved in implantation. Among them are adhesion molecules, including glycosylated cell adhesion molecule 1 (*GLYCAM1*) (Spencer et al., 2007b), galectin 15 (*LGALS15*) (Gray et al., 2004), and secreted phosphoprotein 1 (*SPP1* or osteopontin) (Johnson et al., 2014); transcription factors, including signal transducer and activator of transcriptions (*STATs*) (Stewart et al., 2001) and fibroblast growth factor (*FGF*) (Ocón-Grove et al., 2008); and receptors, including oxytocin receptor (*OXTR*) (Spencer et al., 2007a) and estrogen receptor α (*ESR1*) (Spencer et al., 2007a). Besides the protein-coding genes in the whole transcriptome of mammals, several non-coding genes (ncRNAs) have also been identified, especially the long non-coding RNAs (lncRNAs) (Xu et al., 2016; Zhang et al., 2016). lncRNAs about 200 bp to >100 kb long have been identified as the most abundant ncRNA families (Xu et al., 2016). Indeed, a considerable number of lncRNAs are transcribed by RNA polymerase II, which is similar to mRNAs without the protein-coding ability or formed by processing other transcripts (Plewka et al., 2018). Following the recently reported findings, lncRNAs have received substantial attention owing to their functions in genomic imprinting, epigenetic regulation, and transcriptional control (Kretz et al., 2012). Meanwhile, with high-throughput sequencing, an increasing number of lncRNAs in the endometrium have been characterized in species, such as humans (Chen et al., 2018), pigs (Wang et al., 2016), and rats (Zhang et al., 2018). However, a systematic analysis of lncRNAs expressed in goat endometrium during the “window of implantation” is not yet to be reported.

Strand-specific Ribo-Zero RNA-Seq is eminently employed in evaluating transcriptomes particularly for incompletely annotated genomes, including goats. Herein, we aimed to systematically identify lncRNA and mRNA expression patterns in the endometrial tissue of goat on 16-day pregnancy (“window of implantation”) and the corresponding cyclic controls via this method. As a result, the differentially expressed lncRNAs and mRNAs were identified, where the former was further used in predicting *cis*- and *trans*- target genes. And then, Gene Ontology (GO) and Kyoto Encyclopedia of Genes and Genomes (KEGG) pathways were performed for target genes and mRNAs to significantly determine the changed biological processes (BPs) and pathways by comparing transcriptomic profiles in different pregnancy states. Therefore, this study purposed to obtain an in-depth understanding of endometrial transcriptome changes and present new evidence for the underlying mechanisms of lncRNAs and mRNAs associated with the initiation of preparation for embryo implantation and placentation.

## Materials and Methods

### Ethics Statement and Sample Collection

All the experiments involving animals were conducted under a protocol approved by the Ethics Committees of the Laboratory Animal Center of South China Agricultural University (permit number: SYXK-2014-0136). Six healthy primiparous Chuanzhong black goats (*Capra hircus*) were obtained from Guangdong Wen’s Foodstuffs Group Co., Ltd. (Yunfu, China). The animals were randomly assigned to the cyclic (*n* = 3) and pregnant (*n* = 3) groups. Goats that belong to the pregnant group were artificially inseminated twice after estrus. Goats were slaughtered at a local slaughterhouse on day 16 (*n* = 3) of the estrous cycle (C16) or day 16 (*n* = 3) of pregnancy (P16). The uteri were removed rapidly and transported in an icebox to the laboratory. Pregnancy was confirmed by the presence of apparently normal filamentous conceptuses in uterine flushing (Newton et al., 2019). The endometrial samples were collected and stored at −80°C for RNA extraction.

### RNA Extraction and Library Construction

The total RNA was isolated from each individual sample using TRIzol reagent (Invitrogen, Carlsbad, CA, United States) and following the instructions of the manufacturer. The quality of RNA and concentration were detected by NanoDrop 2000 equipment (Thermo Scientific, Waltham, MA, United States). RNA integrity was assessed using the RNA Nano 6000 Assay Kit of the Bionalyzer 2100 system (Agilent Technologies, CA, United States). A total amount of 3-μg RNA per sample was used as an input material for the RNA sample preparations. First, the Epicenter Ribo-zero^TM^ rRNA Removal Kit (Epicenter, United States) was used to remove ribosomal RNAs, and rRNA free residue was cleaned up by ethanol precipitation. Subsequently, the NEBNext^®^ Ultra^TM^ Directional RNA Library Prep Kit (NIB, United States) was used to generate the sequencing library by using rRNA-depleted RNA according to the recommendations of the manufacturer. Briefly, fragmentation was carried out using divalent cations under elevated temperature in NEBNext First Strand Synthesis Reaction Buffer (5×). The random hexamer primer and M-MuLV Reverse Transcriptase (RNaseH-) were utilized to synthesize the first strand cDNA. Afterward, a second strand cDNA synthesis was performed using DNA Polymerase I and RNase H. In the reaction buffer, dNTPs with dTTP were replaced by dUTP. The remaining overhangs were converted into blunt ends via exonuclease/polymerase activities. Following the adenylation of 3’ ends of DNA fragments, a NEBNext adaptor with hairpin loop structure was ligated to prepare for hybridization. The library fragments were purified with AMPure XP system to obtain 150–200 bp cDNA fragments preferentially (Beckman Coulter, Beverly, CA, United States). Thereafter, a 3-μl USER enzyme (NEB, United States) was used with size-selected, adaptor-ligated cDNA at 37°C for 15 min and was followed by another 5 min at 95°C before PCR. Thereafter, PCR was performed by using Index (X) Primer, Universal PCR primers, and Phusion high-fidelity DNA polymerase. Finally, products were purified by the AMPure XP system, and the library quality was assessed with the Agilent Bioanalyzer 2100 system.

### Sequencing and Quality Control

TruSeq PE Cluster Kit v3-cBot-HS (Illumina) was used to cluster the index-coded samples on a cBot cluster generation system as per the manufacturer’s instructions. After cluster generation, the libraries were sequenced on an Illumina NovaSeq 6000 platform at Novogene (Beijing, China), and 125 bp paired-end reads were produced. The raw reads in fastq format were initially processed through internal Perl scripts. During this step, clean reads were obtained by removing reads that contain adapter, low-quality reads, ploy-N from raw data. Simultaneously, the GC content, Q20, and Q30 of the clean data were calculated. All the downstream bioinformatics analyses were based on high-quality clean data.

### Transcriptome Assembly and Coding Potential Analysis

Reference genome^1^ and gene model annotation files^2^ were directly downloaded from genome website. HISAT2 (v2.0.4, chain specific parameters: –rna-strandness RF) was used to build the index of the reference genome whereas the paired-end clean reads were aligned to the reference genome by HISAT2 v2.0.4 (Langmead and Salzberg, 2012). The reads map of each sample was assembled by StringTie (v1.3.3) through a reference-based approach (Pertea et al., 2016). The novel network process algorithms and optional reassembly steps were performed by StringTie to assemble and quantify transcripts representing multiple splice variants for each gene locus.

The following steps were carried out based on the structural characteristics and non-coding function of lncRNA to obtain high-quality lncRNA. First, transcripts having < 2 exon numbers, low credible single-exon transcripts, and low expression levels, were filtered out. Second, transcripts < 200 bp were filtered out. Third, the above transcripts were annotated through Cuffcompare software. Fourth, the expression level of each transcript was calculated by Cuffquant, while the transcript of FPKM < 0.5 was removed. Lastly, the encoding potential screening employs three coding potential software, namely, CNCI (v2) (Liang et al., 2013), CPC2(v0.1) (Kang et al., 2017), and Pfam-scan (v1.3) (Bateman et al., 2000; Finn et al., 2014) with default parameters, were used to screen the transcripts. The transcripts without coding potential on the above three software were considered candidate lncRNAs.

### Differential Expression Analysis

StringTie (v2.1.1) was used on each sample to calculate fragments per kilo-base of exon per million fragments (FPKMs) of lncRNAs and coding genes (Pertea et al., 2016). The edgeR, a Bioconductor software package for examining the differential expression of replicated count data, was used to determine the differential expression (Robinson et al., 2010). Transcripts with *p*-adjust < 0.05 using Benjamini–Hochberg were considered differentially expressed.

### Target Gene Prediction of the LncRNAs

For the *cis* role of target gene prediction, we searched the coding genes of 100k upstream and downstream of lncRNA and subsequently analyzed their function. However, the *trans* role of target gene prediction was calculated from the expressed correlation between lncRNAs and coding genes with R function “cor” and “cor.test.” The function “cor” was used to calculate the correlation coefficient between lncRNAs and coding genes whereas the function “cor.test” was used to calculate the *p*-value of the correlation coefficient. On the co-expressed genes, the default parameters of miRanda (v 3.3) software were used to further predict the target relationship between co-expression genes and lncRNAs (Enright et al., 2003).

### GO and KEGG Enrichment Analysis

The GO enrichment analysis of differentially expressed mRNAs or lncRNAs was performed by the GOseq R package (Young et al., 2010). GO terms with corrected *p*-value < 0.05 were considered significantly enriched by differentially expressed genes, which used Benjamini–Hochberg to adjust the *p*-value. KEGG (Kanehisa et al., 2007) is a database for understanding high-level functions and utilities of the biological system, such as the cell, organism, and ecosystem, from molecular-level information, particularly large-scale molecular datasets generated by genome sequencing^3^. We used KOBAS software (Mao et al., 2005) to examine the statistical enrichment of the related genes.

### Validation of Gene Expression via Quantitative RT-PCR

The RNA samples from six animals were analyzed by quantitative PCR (RT-qPCR). The reverse transcriptase kit with a gDNA eraser (TaKaRa, Dalian) was used to synthesize cDNA. Subsequently, the cDNAs were used for qPCR in the Step One Plus Real Time PCR System (Life Technologies, MD, United States) by using the SYBR Green PCR Master Mix (TaKaRa, China). The procedure of qPCR was performed as follows. Temperature was regulated at 95°C for 5 min, 40 cycles of 95°C for 15 s, 60°C for 20 s, and 72°C for 20 s. Primers (Supplementary Table S1) were designed using Oligo 7, and specifications were confirmed by NCBI BLAST. Goat GAPDH was used as an internal control, and all reactions were run in triplicate. The relative expression levels were calculated with the 2^–ΔΔ*Ct*^ method. One-way ANOVA by SPSS software was employed to compare the differences. Statistical differences were considered significant when *p* < 0.05 (^∗^) and very significant when *p* < 0.01 (^∗∗^).

## Results

### Read Mapping

In this study, we collected three cyclic (C16_1, C16_2, C16_3) and three pregnant (P16_1, P16_2, P16_3) goat endometrial samples. The RNA sequencing was performed on the Illumina NovaSeq 6000 platform and the expression of lncRNA in endometrial samples was detected. The Pearson correlation among the same group samples was between 0.851 and 0.921 (Supplementary Table S2). A total of 579,092,338 paired-end reads were obtained. Moreover, a total of 546,743,676 clean reads were gathered after filtering out the empty adaptor sequences and of low quality. Thereafter, bioinformatics analysis was conducted based on the clean reads. The clean reads obtained in each library accounted for 93.66–94.84% of the raw reads, and the GC and Q30 contents were more than 50.10 and 88.25%, respectively (Table 1). Subsequently, more than 94% of the clean reads were mapped to the *C. hircus* reference genome (Table 1).

**TABLE 1:** Summary of clean reads mapped to the *Capra hircus* reference genome.

Sample	C16_1	C16_2	C16_3	P16_1	P16_2	P16_3
Raw reads	105,481,500	85,309,714	94,943,370	97,864,228	94,808,198	100,685,328
Clean reads	99,629,264	80,904,562	88,927,098	92,771,084	89,489,124	95,022,544
Q30 (%)	89.92	89.73	88.57	88.25	88.33	88.78
GC content (%)	50.57	50.81	51.67	50.69	50.10	50.90
Total mapped	94,684,974 (95.04%)	76,855,661 (95%)	84,228,203 (94.72%)	87,992,780 (94.85%)	84,961,494 (94.94%)	90,487,836 (95.23%)

### Identification and Characteristics of lncRNAs in Goat Endometrium

**FIGURE 1:**
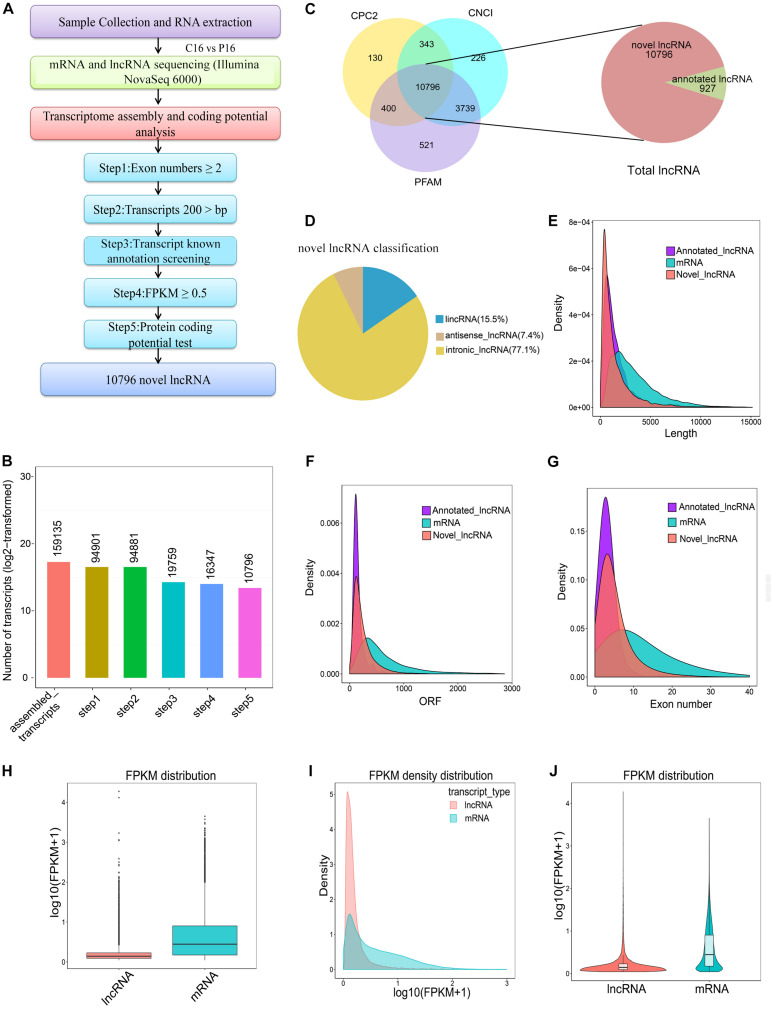
Screening and characterization of endometrial lncRNA. **(A)** Bioinformatics pipeline for endometrial lncRNA discovery. **(B)** A total of 159,135 transcripts were assembled with a stringent filtering pipeline to discard transcripts without all characteristics of lncRNA (see section “Materials and Methods” for specific steps). **(C)** The filtration of the candidate lncRNAs is indicated through Venn diagrams. The coding potential analysis of candidate lncRNAs was performed by using three conventional tools (PFAM, CPC, and CNCI), and 10,796 novel lncRNAs were identified by the three software programs. **(D)** Classification of novel lncRNA. **(E)** Transcript lengths distribution of the lncRNAs and mRNAs. **(F)** ORF length of the lncRNAs and mRNAs. **(G)** Exon number distribution of the lncRNAs and mRNAs. **(H)** Boxplot of expression level [showed in log10 (FPKM + 1)] for lncRNA and mRNAs. **(I)** FPKM density distribution of lncRNA and mRNAs. **(J)** Violin plot of expression level [showed in log10 (FPKM + 1)] for lncRNA and mRNA transcripts.

After certain filtering steps, 16,347 long intron-containing transcripts were assembled through Cuffcompare and Cuffquant to filter out the immature mRNA fragments. Three softwares, namely, Coding-Non-Coding-Index (CNCI), Pfam-scan (PFAM), and Coding Potential Calculator (CPC) were used to remove the potential coding transcripts, hence leading to the identification of a total of 11,723 lncRNAs. Among these lncRNAs, 927 molecules were annotated lncRNAs in goat and 10,796 molecules were novel lncRNAs in goat (Figures 1A–C and Supplementary Table S3). Notably, 15.5, 77.1, and 7.4% of the novel lncRNAs were lincRNAs (long intergenic non-coding RNAs), intronic lncRNAs, and antisense lncRNAs, respectively (Figure 1D). To understand the differences between lncRNAs and mRNAs, the information on gene structure and expression pattern were selected to be compared and analyzed. From the analysis, it was evident that the average length of lncRNA and mRNA was 1590.65 and 3477.17 nt, respectively (Figure 1E). However, the length of the open reading frame (ORF) of lncRNA was also shorter than that of mRNA (Figure 1F). Unlike mRNA, most lncRNAs had few exons. For instance, the average number of exons in the lncRNAs was approximately 5, while that in mRNAs was more than 12 (Figure 1G). Besides, the lncRNA expression level was lower than that of mRNA (Figures 1H–J).

### Differentially Expressed mRNAs and lncRNAs in Goat Endometrium

**FIGURE 2:**
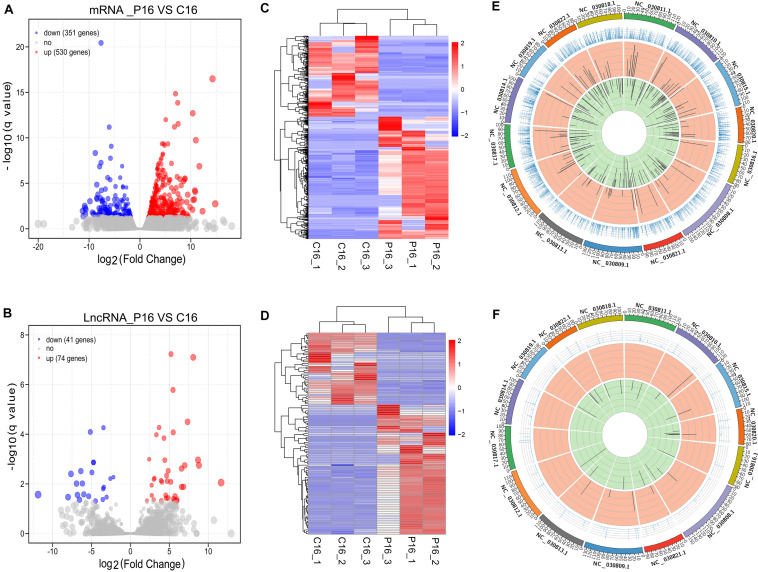
Overall differences of coding genes and lncRNAs in pregnant and non-pregnant endometria. The volcano plots of the differentially expressed mRNAs **(A)** and lncRNAs **(B)**. Heatmap showing the expression profile of mRNAs **(C)** and lncRNAs **(D)**. The top panel is the tree constructed through the Pearson correlation. The Circos diagram depicts the chromosomal distribution of differentially expressed mRNAs **(E)** and lncRNAs **(F)**. The outermost ring represents different chromosomes; the second ring represents the FPKM of transcripts; the third ring represents the upregulated mRNAs or lncRNAs; and the fourth ring represents the downregulated mRNAs or lncRNAs.

Herein, our study proved, a total of 881 mRNAs were differentially expressed in the goat endometrium between C16 and P16. Among these mRNAs, 530 were upregulated while 351 were downregulated in P16 compared with C16 (Figure 2A and Supplementary Table S4). Meanwhile, 115 lncRNAs were differentially expressed in the goat endometrium. Of these lncRNAs, 74 were upregulated whereas 41 were downregulated (Figure 2B and Supplementary Table S4). Unsupervised hierarchical clustering analysis was conducted to analyze the expression patterns of DE mRNAs and lncRNAs on pregnant and non-pregnant conditions. As shown from the heatmap analysis, the expression of the same gene and lncRNAs in the same pregnant condition is almost identical, indicating an extremely small difference between the samples in the same group. Large differences between samples under different pregnancy conditions (Figures 2C,D) were demonstrated through a sample cluster analysis. Distribution of DE transcripts in a chromosome was studied by calculating the density of the total mapped reads against each chromosome. Differential mRNAs were concentrated on chromosomes NC-030810.1, NC-030815.1, NC-030809.1, NC-030808.1, NC-030811.1, NC-030812.1, NC-030817.1, NC-030818.1, and NC-030822.1 (Figure 2E). Notably, all of these chromosomes exhibited more downregulated genes than upregulated genes. As of the differential lncRNAs, they were concentrated on chromosomes NC-030820.1, NC-030808.1, NC-030809.1, and NC-030822.1 (Figure 2F).

### Validation of Sequencing Results by qRT-PCR

**FIGURE 3:**
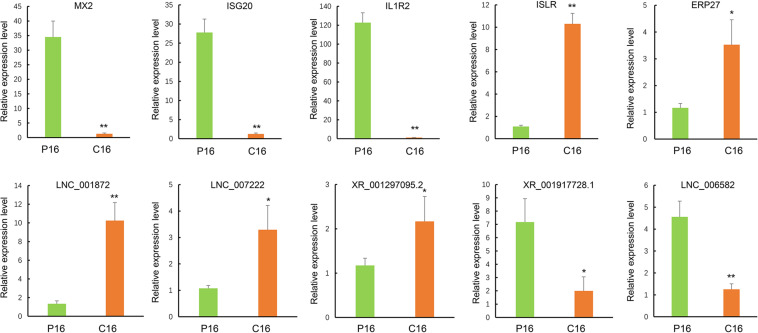
Validation of sequencing results via RT-qPCR. The transcript expression was quantified relative to the expression level of GAPDH by using the comparative cycle threshold (−△△Ct) method. The data are shown as the mean ± SD (*n* = 3), *P < 0.05, **P < 0.01.

To further evaluate the reliability of RNA sequencing, five DE lncRNAs (LNC001872, LNC007222, LNC006582, XR001297095.2, and XR001917728.1) and five DE mRNAs (*MX2*, *ISG20*, *IL1R2*, *ISLR*, and *ERP27*) were randomly selected for validation through qRT-PCR. All levels of DE lncRNAs and mRNAs were significantly different in their expression between the pregnant and non-pregnant groups (Figure 3), thereby indicating that the RNA-seq data were reliable.

### GO and KEGG Enrichment Analysis of the Differential mRNAs

**FIGURE 4:**
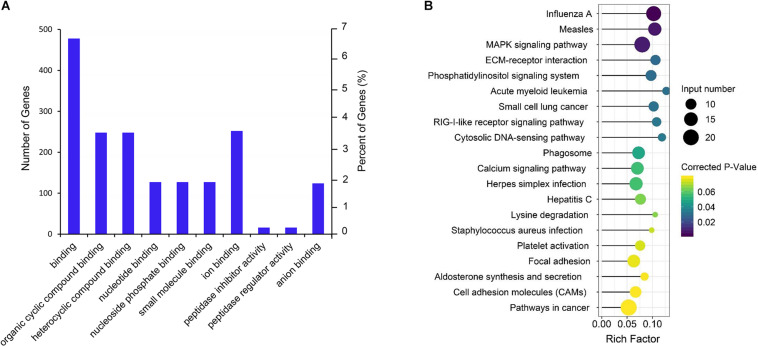
GO and KEGG enrichment analysis of differential expressed mRNAs. **(A)** GO analysis of differentially expressed mRNAs. **(B)** Top 20 KEGG pathways of differentially expressed mRNAs. The *x*-axis indicates the number of unique sequences assigned to a specific pathway and the *y*-axis indicates the KEGG pathway. The size of the circle represents the number of genes enriched in the pathway. The darker the color, the more significant the enrichment.

Gene Ontology term and KEGG enrichment analyses were performed to ascertain the functions of the differentially expressed mRNAs and their association. The significantly enriched GO terms were only distributed in MF (Figure 4A and Supplementary Table S5). Among the top 10 MF terms, seven were mainly associated with binding to key molecules, which were similar to the GO results of the co-expression genes of differential lncRNAs. A potential strong connection and interaction between mRNAs and lncRNAs were evident from the results. However, the KEGG pathway analysis of the differentially expressed mRNAs revealed a significant portion of pathways related to reproduction. Among the top 20 enriched pathways includes MAPK signaling pathway, ECM–receptor interaction, phosphatidylinositol signaling system, RIG-I-like receptor signaling pathway, focal adhesion, aldosterone synthesis, and secretion and cell adhesion molecules (CAMs) (Figure 4B and Supplementary Table S6).

### Differentially Expressed lncRNAs Target Genes Prediction

Given that lncRNAs have no encoding potential, their functions are achieved through regulation of target genes. Herein, we predicted the potential targets of lncRNAs via *cis* (co-location) and *trans* (co-expression) regulation to explore the possible function of the differentially expressed lncRNAs. Among the 115 differential lncRNAs transcripts, 108 corresponded to 445 related mRNA transcripts based on co-location analysis. Furthermore, 115 lncRNA transcripts corresponded to 9153 related mRNA transcripts based on co-expression analysis.

### The GO and KEGG Enrichment Analysis of the Co-located Genes of Differential lncRNAs

**FIGURE 5:**
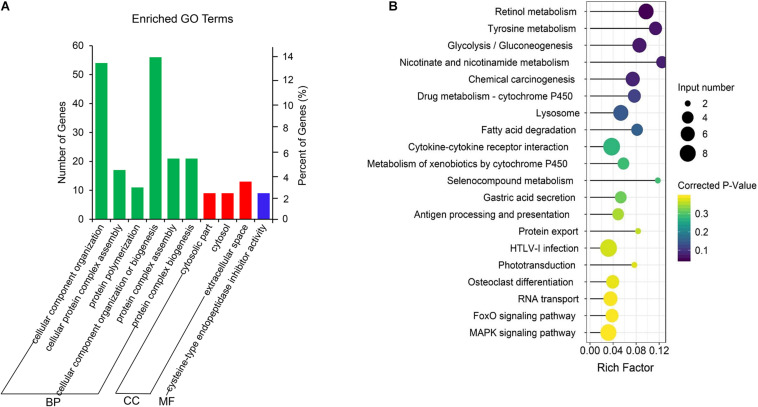
GO and KEGG enrichment analysis of co-located genes of differential expressed lncRNAs. **(A)** GO analysis of related gene of differential lncRNAs. BP, biological process; CC, cell composition; MF, molecular function. **(B)** Top 20 KEGG pathways of related gene of differential lncRNAs. The *x*-axis indicates the number of unique sequences assigned to a specific pathway and the *y*-axis indicates the KEGG pathway. The size of the circle represents the number of genes enriched in the pathway. The darker the color, the more significant the enrichment.

The co-located genes of differential lncRNAs were used for the GO and KEGG enrichment analyses. Significantly (*q* < 0.05) enriched GO terms in BP, cellular component (CC), and molecular function (MF) are highlighted in Figure 5A. Only 10 significantly enriched GO terms results were obtained, a majority were associated with protein synthesis and degradation. These synthesis and degradation processed were related to endometrium remodeling, involving cysteine-type endopeptidase inhibitor activity, cellular protein complex assembly, protein polymerization, protein complex assembly, and protein complex biogenesis. Findings from the present study suggest that these GO terms share common genes. Two common genes, HELZ and KRT17, were differentially expressed. The lncRNAs that co-localized with the two genes were LNC_007472 and LNC_007827. Figure 5B demonstrates the KEGG enrichment result (Supplementary Table S6). The most significant signal pathway is Retinol metabolism while the second significant signal pathway is Tyrosine metabolism. Five common genes among the two pathways are evident, including ADH4, ADH6, ADH7, LOC102181105, and Aox1. These related genes co-localized with XR_001918173.1, LNC_002760, and LNC_000599. Also, certain reproductive related genes, such as CST3, TGFB1, LGALS15, CST6, CXCL10, FOXS1, GRP and RSAD2, were detected located near the LNC_005256, LNC_006979, LNC_007223, LNC_010092, LNC_002677, LNC_005318, LNC_009053, and LNC_004709 (Table 2).

**TABLE 2:** Genomic association between lncRNAs and nearby reproductive related genes.

Transcript_ID	FPKM (P16/C16)	Q value	mRNA	FPKM (P16/C16)	Q value	Distance	Location	Function of mRNA
LNC_005256	120.93/22.69	1.02E-03	CST3	1802.78/351.19	n.s	−3956	Antisense	Modifies the glycocalyx on endometrial epithelium cells and trophectoderm during implantation (Carson et al., 2000)
LNC_006979	1.38/0.00	2.14 E-04	TGFB1	0.94/1.01	n.s	86,383	Upstream	Plays an important role in embryo implantation by activating the SMAD pathway (Vineet Kumar et al., 2013)
NC_007223	21.81/0.48	8.54 E-05	LGALS15	3284.11/575.29	2.19E-03	−1777	Antisense	Regulates trophectoderm adhesion and proliferation (Gray et al., 2004)
LNC_010092	20.39/0.95	1.11 E-03	CST6	163.84/8.95	7.40E-07	−1470	Antisense	Plays a key role in endometrial remodeling during the establishment of pregnancy (Shim et al., 2013)
LNC_002677	4.33/0.16	7.76 E-03	CXCL10	344.84/15.15	5.93E-09	−2326	Antisense	It is implicated in the establishment of an immune tolerant environment for the embryo (Złotkowska and Andronowska, 2019)
LNC_005318	0.767/0.05	4.85 E-02	FOXS1	24.81/4.43	1.10E-03	1952	Upstream	Plays a role as a negative feedback regulator of IFNT signaling in bovine endometrial epithelial cells (Kusama et al., 2017)
LNC_009053	70.01/10.43	2.91 E-02	GRP	604.98/97.44	1.27 E-02	−12,976	Antisense	IFNT-stimulated gene that can affect the cell proliferation and migration of the ovine uterus (Gwonhwa et al., 2008)
LNC_004709	9.87/0.27	5.93E-08	RSAD2	474.66/14.07	1.00E-12	−18,542	Antisense	Regulates implantation through the modulation of local immune cells in the endometrium (Song et al., 2007)

*Note: FPKM, fragments per kilo base of exon per million fragments mapped; n.s, no significance.*

### GO and KEGG Enrichment Analysis of the Co-expression Genes of Differential lncRNAs

**FIGURE 6:**
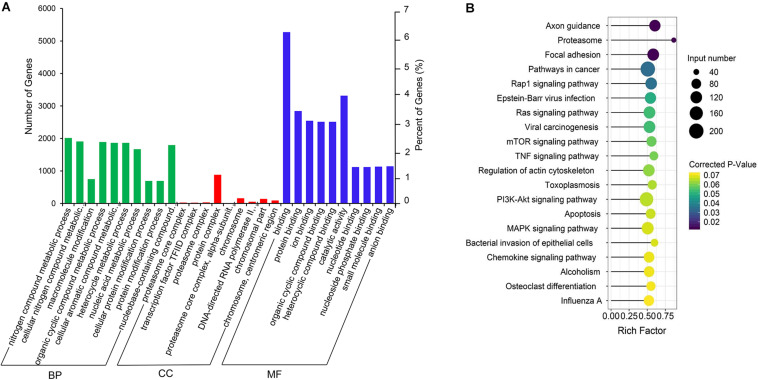
GO and KEGG enrichment analysis of co-expressed genes of differentially expressed lncRNAs. **(A)** GO analysis of related gene of differential lncRNAs. BP, biological process; CC, cell composition; MF, molecular function. **(B)** Top 20 KEGG pathways of the related gene of differential lncRNAs. The *x*-axis indicates the number of unique sequences assigned to a specific pathway and the *y*-axis indicates the KEGG pathway. The size of the circle represents the number of genes enriched in the pathway. The darker the color, the more significant the enrichment.

**FIGURE 7:**
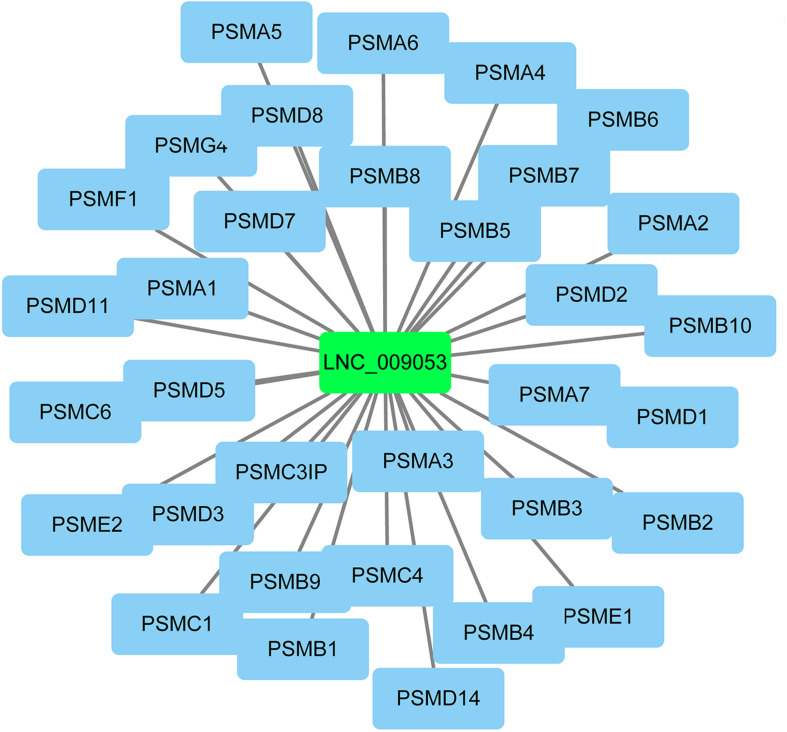
Potential targets related to proteasome pathway of lnc_009053.

**FIGURE 8:**
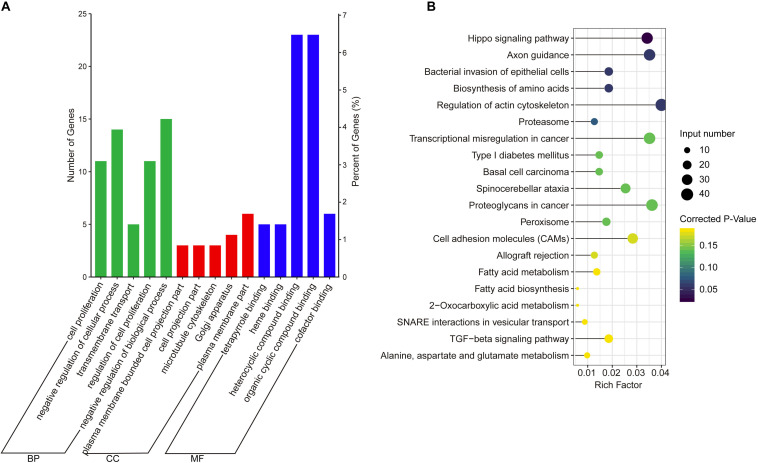
GO and KEGG enrichment analysis of target genes of differential expressed lncRNAs. **(A)** GO analysis of related gene of differential lncRNAs. BP, biological process; CC, cell composition; MF, molecular function. **(B)** Top 20 KEGG pathways of related gene of differential lncRNAs. The *x*-axis indicates the number of unique sequences assigned to a specific pathway and the *y*-axis indicates the KEGG pathway. The size of the circle represents the number of genes enriched in the pathway. The darker the color, the more significant the enrichment.

The co-expression genes of differential lncRNAs were used for the GO and KEGG enrichment analyses. The top 10 significantly (*q* < 0.05) enriched GO terms in BP, CC, and MF are listed in Figure 6A, while all the GO enrichment terms are shown in Supplementary Table S5. Interestingly, similar to the GO results of the co-located genes of differential lncRNAs, a significant portion of GO terms in BP and CC were related to protein modification, synthesis, and degradation, such as cellular protein modification process, protein modification process, proteasome core complex, transcription factor TFIID complex, proteasome complex, protein complex, and proteasome core complex. Moreover, the top 10 enriched terms in MF were mainly associated with binding of key molecules, such as protein binding, ion binding, organic cyclic compound binding, heterocyclic compound binding, nucleotide binding, nucleoside phosphate binding, small molecule binding, and anion binding. KEGG pathway analysis showed that several pathways were related to endometrium development, for example, proteasome, focal adhesion, rap1 signaling pathway, ras signaling pathway, and mTOR signaling pathway (Figure 6B and Supplementary Table S6). Therefore, GO terms and KEGG pathway likewise focuses on the proteasome, which is related to protein degradation. We subsequently screened the differential lncRNAs co-expressed with the genes of the proteasome. Notably, LNC_009053 might target 33 genes of proteasome via co-expression regulation (Figure 7), thereby revealing the potential to regulate endometrium remolding through the proteasome pathway. We further analyzed the target relationships between these co-expressed genes and differential lncRNAs, and screened out a total of 2339 genes with target relationships between lncRNAs (Supplementary Table S7). These genes were subjected to GO enrichment and KEGG pathway analysis. The BPs including cell proliferation, transmembrane transport, and regulation of cell proliferation which are related to the endometrium development were in the top five BP terms of GO enrichment (Figure 8A and Supplementary Table S5). KEGG pathway analysis also showed that some pathways were related to endometrial remodeling and development, such as bacterial invasion of epithelial cells, biosynthesis of amino acids, and regulation of actin cytoskeleton (Figure 8B and Supplementary Table S6).

## Discussion

LncRNAs are transcriptionally regulated and play a significant role in various biological activities, such as cell signaling, cell differentiation, transcriptional regulation, and epigenetics (Cao, 2014; Mathieu et al., 2014; Li, 2016). Based on the current understanding, lncRNAs are associated with the mammalian reproductive system (Wang et al., 2016; Sigurgeirsson et al., 2017; Chen et al., 2018; Zhang et al., 2018). Hence, our study identified and analyzed the lncRNAs in endometrial tissue of goat on 16-day pregnancy and corresponding cyclic controls by RNA-seq. From a total of 11,723 lncRNAs identified, 115 were differentially expressed. To the best of our knowledge, the present study is the first systematic assessment on the lncRNAs in goat endometrium. Furthermore, it is the first to identify differentially expressed mRNAs and lncRNAs between pregnant and cyclic endometrium.

In the study, the endometrial transcriptome of mRNAs and lncRNAs were provided. Pearson’s correlation among the samples in similar groups was between 0.851 and 0.921, revealing a reasonable sample selection. Based on the composition analysis and base quality, the GC content was 50.10–50.90% whereas the Q30 was 88.25–89.92%, thereby indicating that high-quality libraries were created and the quality of the sequencing was good. The sequencing results revealed that the identified lncRNAs in goat endometrium have similar features to those reported in previous studies (Stewart et al., 2001; Macklon et al., 2006; Pauli et al., 2012). Particularly, they were lower in exon number, shorter in average length, short in the average length of putative ORFs, and lower in expression level than protein-coding genes. In our study, we excluded the transcripts having protein-coding potential using three coding potential software (CNCI, CPC2, and Pfam-scan) (Mistry et al., 2007; Liang et al., 2013; Kang et al., 2017). It is worth mentioning that some novel lncRNAs are potential protein-coding mRNAs with unknown protein domains, especially for the novel lncRNAs having long putative ORF (≥500 nt).

LncRNAs rarely encode a protein and hence achieve their functions through regulation of target genes. Numerous studies have shown that the biological functions of lncRNAs can be predicted by assessing relevant genes via *trans-* and *cis*-regulation (Ding et al., 2018; Liu et al., 2018). *Trans* effect is defined as the interaction between lncRNAs and the co-expressing mRNAs, whereas *cis*, as the regulatory action of lncRNAs on genes located downstream or upstream. Given these definitions, 445 *cis*-target mRNAs were identified within 10 kb upstream and downstream of the differential lncRNAs, and 9153 *trans*-target mRNAs co-expressed with lncRNAs were obtained. These genes were further used for lncRNA functional annotation and enrichment analysis. Interestingly, numerous genes, which could play critical roles in goat endometrium during implantation, were co-located with DE lncRNAs (Table 2). For example, the LNC_007223 paired with LGALS15. Of note, LGALS15 is induced by progesterone and IFN-τ in LE/sGE and promotes conceptus implantation by regulating trophectoderm proliferation and adhesion during the implantation phase (Gray et al., 2004; Spencer et al., 2004a). The LNC_007223 co-located with LGALS15 may affect the LGALS15 expression and therefore, involved in the interaction between the conceptus and endometrium. Besides, LNC_005256 and LNC_010092 were co-located with CST3 and CST6, respectively. Both CST3 and CST6 are Cathepsins inhibitors, which are peptidases that can regulate endometrial and trophoblast invasion through degrading extracellular matrix and intracellular proteins (Afonso et al., 1997). However, the expression levels of CST6 and the co-located lncRNA (LNC_010092) in endometrium were significantly higher in P16 than those in C16. With tissue remodeling function, the higher expression of CST6 and LNC_010092 at the window of implantation further indicated their function in promoting embryo implantation. Moreover, many lncRNAs co-expressed with genes, which could play important roles in embryo implantation, were present. For instance, LNC_009053 matched 33 genes of proteasome via co-expression regulation, thus can regulate endometrium remolding by altering the expression of proteasomes.

GO and KEGG enrichment analyses were used to further analyze the function of differentially expressed lncRNAs. The GO analysis of DE lncRNAs (co-location genes) indicated that the terms about protein synthesis were the most significant GO terms, such as cysteine-type endopeptidase inhibitor activity, cellular protein complex assembly, protein polymerization, protein complex assembly, and protein complex biogenesis. Similar to cattle and other ruminants, goat implantation occurs as a protracted process. That is, the maternal recognition of pregnancy occurs at about 15–17 days of pregnancy. Subsequently, the adhesion of trophectoderm and endometrial epithelium occurs at 19–23 days of pregnancy. During this period, the developing conceptus undergoes dramatic morphological changes and migrates throughout the entire uterine lumen (Gnatek et al., 1989; Wango et al., 1990). To support conceptus survival, growth, and development, the maternal endometrium should synthesize a large amount of nutrients, such as amino acids, glucose, minerals, and enzymes, and secrete them into the uterine lumen as term histotroph (Gray et al., 2002; Bazer et al., 2012). The discussion above implies that the high activity of protein synthesis is necessary for embryo implantation. The KEGG enrichment analysis of DE lncRNAs (co-location genes) indicates that retinol metabolism is the most significant signal pathway. Retinol (alcohol form vitamin A), as an essential nutrient, plays vital roles in the developing notochord and nervous system and other embryonic structures, as well as in the maintenance of immune competence, epithelial surfaces, and reproduction (Rune and Heidi Kiil, 2010). As goats possess a long period of peri-implantation, large amount of nutrients, including vitamin A, must be provided by maternal endometrium to meet the rapid cellular growth for conceptuses development. Given the high degree of hydrophobicity, vitamin A needs the specific carrier protein (retinol-binding protein, *RBP*) to be solubilized and transported in the body fluids (Bellovino et al., 2003). Consistently, RBP is highly expressed in caprine endometrium during the peri-implantation period of pregnancy (Liu et al., 2003). Therefore, the co-location genes of the differentially expressed lncRNAs may be mainly related to the synthesis of amino acids, vitamin A, and other nutrients, which are necessary for the rapid development of the embryo. The GO and KEGG analyses of co-expression genes of differentially expressed lncRNAs indicated that three terms about proteasome were significantly enriched, including proteasome core complex, proteasome complex, and proteasome core complex, alpha-subunit complex. Furthermore, the pathway of the proteasome was the second significant signal pathway. As a result, these data proved that the co-expression genes of the differentially expressed lncRNAs could regulate endometrial remodeling by affecting the expression of genes of proteasomes. Previous studies have demonstrated that endometrial remodeling occurs during the implantation period and plays critical roles in trophoblast invasion and implantation (Forde et al., 2011; Bazer, 2013). Combining the functional analysis of co-expressed and co-localized genes of lncRNAs, we propose that differentially expressed lncRNAs may be mainly related to the nutrient synthesis and endometrial remodeling. Hence, the network analysis of co-location and co-expression is merely a speculation, and the results still require the support of experimental data.

## Conclusion

In summary, we conducted genome-wide RNA-seq for goat endometrium on 16-day pregnancy or estrous cycle and identified mRNA and lncRNA expression profiles. Importantly, the genomic structures, differential expression, and target pathways of differentially expressed lncRNAs were elucidated. Functional annotations revealed that certain lncRNAs were related to key BPs and pathways associated with embryo implantation. However, further studies should focus on elucidating specific molecular mechanisms of the candidate lncRNAs in endometrial receptivity.

## Data Availability Statement

The datasets presented in this study can be found in online repositories. The names of the repository/repositories and accession number(s) can be found below: https://www.ncbi.nlm.nih.gov/, PRJNA578518.

## Ethics Statement

The animal study was reviewed and approved by the Ethics Committee of the Laboratory Animal Center of South China Agricultural University. Written informed consent was obtained from the owners for the participation of their animals in this study.

## Author Contributions

LJH, QH, GBL, and DL designed the study. XPZ, YSX, CZ, XZ, and YKL collected and treated the goat endometrium. QH, GBL, XZ, MD, and YQG conducted the experiments and performed the sequencing analysis. QH, GBL, DL, and LJH drafted the manuscript. All authors read and approved the final manuscript.

## Conflict of Interest

The authors declare that the research was conducted in the absence of any commercial or financial relationships that could be construed as a potential conflict of interest.

## Footnotes

1 ftp://ftp.ncbi.nlm.nih.gov/genomes/all/GCF/001/704/415/GCF_001704415.1_ARS1/GCF_001704415.1_ARS1_genomic.fna.gz

2 ftp://ftp.ncbi.nlm.nih.gov/genomes/all/GCF/001/704/415/GCF_001704415.1_ARS1/GCF_001704415.1_ARS1_genomic.gff.gz

3 http://www.genome.jp/kegg/

## References

[B1] Afonso S. Romagnano L. Babiarz B. (1997). The expression and function of cystatin C and cathepsin B and cathepsin L during mouse embryo implantation and placentation. *Development* 124 3415–3425. 9310336 10.1242/dev.124.17.3415

[B2] Ai-Guo S. Ji-Long L. Xiao-Ming J. Jian-Zhi R. Cai-Hui M. Wei L. (2011). Genome-wide identification of micro-ribonucleic acids associated with human endometrial receptivity in natural and stimulated cycles by deep sequencing. *Fertil. Steril.* 96 150–155. 10.1016/j.fertnstert.2011.04.072 21601191

[B3] Altmäe S. Martinez-Conejero J. A. Esteban F. J. Ruiz-Alonso M. Stavreus-Evers A. Horcajadas J. A. (2013). MicroRNAs miR-30b, miR-30d, and miR-494 regulate human endometrial receptivity. *Reprod. Sci.* 20 308–317. 10.1177/1933719112453507 22902743 PMC4077381

[B4] Bateman A. Birney E. Durbin R. Eddy S. R. Howe K. L. Sonnhammer E. L. L. (2000). The Pfam protein families database. *Nucleic Acids Res.* 28 263–266. 10.1093/nar/28.1.263 10592242 PMC102420

[B5] Bazer F. Satterfield M. Song G. (2012). Modulation of uterine function by endocrine and paracrine factors in ruminants. *Anim. Reprod.* 9 305–311.

[B6] Bazer F. W. (2013). Pregnancy recognition signaling mechanisms in ruminants and pigs. *J. Anim. Sci. Biotechnol.* 4 23–23. 23800120 10.1186/2049-1891-4-23 PMC3710217

[B7] Bazer F. W. Spencer T. E. Ott T. L. (2011). Interferon tau: a novel pregnancy recognition signal. *Am. J. Reprod. Immunol.* 37 412–420. 10.1111/j.1600-0897.1997.tb00253.x 9228295

[B8] Bazer F. W. Thatcher W. W. Hansen P. J. Mirando M. A. Ott T. L. Plante C. (1991). Physiological mechanisms of pregnancy recognition in ruminants. *J. Reprod. Fertil. Suppl.* 43 39–47. 1843350

[B9] Bellovino D. Apreda M. Gragnoli S. Massimi M. Gaetani S. (2003). Vitamin A transport: in vitro models for the study of RBP secretion. *Mol. Aspects Med.* 24 411–420. 10.1016/s0098-2997(03)00037-2 14585312

[B10] Cao J. (2014). The functional role of long non-coding RNAs and epigenetics. *Biol. Proced. Online* 16:11. 10.1186/1480-9222-16-11 PMC4177375 25276098

[B11] Carson D. D. Bagchi I. Dey S. K. Enders A. C. Fazleabas A. T. Lessey B. A. (2000). Embryo implantation. *Dev. Biol.* 223 217–237. 10882512 10.1006/dbio.2000.9767

[B12] Chen M. Y. Liao G. D. Zhou B. Kang L. N. He Y. M. Li S. W. (2018). Genome-wide profiling of long noncoding RNA expression patterns in women with repeated implantation failure by RNA sequencing. *Reprod. Sci.* 26 18–25. 10.1177/1933719118756752 29495908

[B13] D’Hauterive S. P. Charlet-Renard C. Goffin F. Foidart M. Geenen V. (2002). The implantation window. *J. Gynécol. Obstét. Biol. Reprod.* 31 440–455. 12379828

[B14] Ding C. Ma J. Yang L. Lin K. Jiang X. Yang Q. (2018). Analysis of long non-coding RNA expression profiles using RNA sequencing in ovarian endometriosis. *Gene* 673 140–148. 10.1016/j.gene.2018.06.046 29920364

[B15] Enright A. J. John B. Gaul U. Tuschl T. Sander C. Marks D. S. (2003). MicroRNA targets in *Drosophila*. *Genome Biol.* 5:R1. 10.1186/gb-2003-5-1-r1 PMC395733 14709173

[B16] Finn R. D. Alex B. Jody C. Penelope C. Eberhardt R. Y. Eddy S. R. (2014). Pfam: the protein families database. *Nucleic Acids Res.* 42 222–230. 10.1093/nar/gkt1223 PMC3965110 24288371

[B17] Forde N. Carter F. Spencer T. E. Bazer F. W. Sandra O. Mansouri-Attia N. (2011). Conceptus-induced changes in the endometrial transcriptome: how soon does the cow know she is pregnant? *Biol. Reprod.* 85 144–156. 10.1095/biolreprod.110.090019 21349821

[B18] Gnatek G. G. Smith L. D. Duby R. T. Godkin J. D. (1989). Maternal recognition of pregnancy in the goat: effects of conceptus removal on lnterestrus intervals and characterization of conceptus protein production during early pregnancy. *Biol. Reprod.* 41 655–663. 10.1095/biolreprod41.4.655 2620074

[B19] Gray C. Burghardt R. Johnson G. Bazer F. Spencer T. (2002). Evidence that absence of endometrial gland secretions in uterine gland knockout ewes compromises conceptus survival and elongation. *Reproduction* 124 289–300. 10.1530/reprod/124.2.289 12141942

[B20] Gray C. A. Adelson D. L. Bazer F. W. Burghardt R. C. Meeusen E. N. T. Spencer T. E. (2004). Discovery and characterization of an epithelial-specific galectin in the endometrium that forms crystals in the trophectoderm. *Proc. Natl. Acad. Sci. U.S.A.* 101 7982–7987. 10.1073/pnas.0402669101 15148380 PMC419543

[B21] Gwonhwa S. Satterfield M. C. Jinyoung K. Bazer F. W. Spencer T. E. (2008). Gastrin-releasing peptide (GRP) in the ovine uterus: regulation by interferon tau and progesterone. *Biol. Reprod.* 79 376–386. 10.1095/biolreprod.108.068403 18448839 PMC2714990

[B22] Hansen T. R. Austin K. J. Perry D. J. Pru J. K. Teixeira M. G. Johnson G. A. (1999). Mechanism of action of interferon-tau in the uterus during early pregnancy. *J. Reprod. Fertil. Suppl.* 54 329–339. 10692865

[B23] Johnson G. A. Burghardt R. C. Bazer F. W. (2014). Osteopontin: a leading candidate adhesion molecule for implantation in pigs and sheep. *J. Anim. Sci. Biotechnol.* 5:56. 10.1186/2049-1891-5-56 PMC4322467 25671104

[B24] Kanehisa M. Araki M. Goto S. Hattori M. Hirakawa M. Itoh M. (2007). KEGG for linking genomes to life and the environment. *Nucleic Acids Res.* 36(Suppl._1), D480–D484. 18077471 10.1093/nar/gkm882 PMC2238879

[B25] Kang Y. J. Yang D. C. Kong L. Hou M. Meng Y. Q. Wei L. (2017). CPC2: a fast and accurate coding potential calculator based on sequence intrinsic features. *Nucleic Acids Res.* 45 W12–W16. 28521017 10.1093/nar/gkx428 PMC5793834

[B26] Kretz M. Dan E. W. Flockhart R. J. Lee C. S. Zehnder A. Lopezpajares V. (2012). Suppression of progenitor differentiation requires the long noncoding RNA ANCR. *Genes Dev.* 26 338–343. 10.1101/gad.182121.111 22302877 PMC3289881

[B27] Kusama K. Bai R. Nakamura K. Okada S. Yasuda J. Imakawa K. (2017). Endometrial factors similarly induced by IFNT2 and IFNTc1 through transcription factor FOXS1. *PLoS One* 12:e0171858. 10.1371/journal.pone.0171858 28199372 PMC5310909

[B28] Langmead B. Salzberg S. L. (2012). Fast gapped-read alignment with Bowtie 2. *Nat. Methods* 9 357–359. 10.1038/nmeth.1923 22388286 PMC3322381

[B29] Li S. (2016). Expression of concern: the functional role of long non-coding RNAs and epigenetics. *Biol. Proced. Online* 18:12. 10.1186/s12575-016-0042-1 PMC4901470 27293384

[B30] Liang S. Haitao L. Dechao B. Guoguang Z. Kuntao Y. Changhai Z. (2013). Utilizing sequence intrinsic composition to classify protein-coding and long non-coding transcripts. *Nucleic Acids Res.* 41:e166. 10.1093/nar/gkt646 23892401 PMC3783192

[B31] Liu K. H. Huang J. C. Lin J. H. (2003). Identification of retinol-binding protein produced by caprine endometrium during periattachment period of early pregnancy. *Asian Australa. J. Anim. Sci.* 15 1708–1713. 10.5713/ajas.2002.1708

[B32] Liu Y. Qi B. Xie J. Wu X. Ling Y. Cao X. (2018). Filtered reproductive long non-coding RNAs by genome-wide analyses of goat ovary at different estrus periods. *BMC Genomics* 19:866. 10.1186/s12864-018-5268-7 30509164 PMC6278114

[B33] Macklon N. S. Stouffer R. L. Giudice L. C. Fauser B. C. (2006). The science behind 25 years of ovarian stimulation for in vitro fertilization. *Endocr. Rev.* 27 170–207. 10.1210/er.2005-0015 16434510

[B34] Mao X. Tao C. Olyarchuk J. G. Wei L. (2005). Automated genome annotation and pathway identification using the KEGG Orthology (KO) as a controlled vocabulary. *Bioinformatics* 21 3787–3793. 10.1093/bioinformatics/bti430 15817693

[B35] Mathieu E. L. Belhocine M. Dao L. T. Puthier D. Spicuglia S. (2014). [Functions of lncRNA in development and diseases]. *Med. Sci.* 30 790–796. 10.1051/medsci/20143008018 25174757

[B36] Mistry J. Bateman A. Finn R. D. (2007). Predicting active site residue annotations in the Pfam database. *BMC Bioinformatics* 8:298. 10.1186/1471-2105-8-298 17688688 PMC2025603

[B37] Newton G. Lewis S. Avendano J. Williams E. Ribeiro F. Nuti L. (2019). Fucosyltransferase gene expression in goat endometrium during the estrous cycle and early pregnancy. *Theriogenology* 132 118–127. 10.1016/j.theriogenology.2019.04.022 31022601

[B38] Ocón-Grove O. M. Cooke F. N. T. Alvarez I. M. Johnson S. E. Ott T. L. Ealy A. D. (2008). Ovine endometrial expression of fibroblast growth factor (FGF) 2 and conceptus expression of FGF receptors during early pregnancy. *Domest. Anim. Endocrinol.* 34 135–145. 10.1016/j.domaniend.2006.12.002 17223006

[B39] Pan Q. Luo X. Toloubeydokhti T. Chegini N. (2007). The expression profile of micro-RNA in eutopic and ectopic endometrium and the influence of ovarian steroids on their expression in endometrial cells. *Mol. Hum. Reprod.* 88 797–806. 10.1093/molehr/gam063 17766684

[B40] Pauli A. Valen E. Lin M. F. Garber M. Vastenhouw N. L. Levin J. Z. (2012). Systematic identification of long noncoding RNAs expressed during zebrafish embryogenesis. *Genome Res.* 22 577–591. 10.1101/gr.133009.111 22110045 PMC3290793

[B41] Pertea M. Kim D. Pertea G. M. Leek J. T. Salzberg S. L. (2016). Transcript-level expression analysis of RNA-seq experiments with HISAT, StringTie and Ballgown. *Nat. Protoc.* 11 1650–1667. 10.1038/nprot.2016.095 27560171 PMC5032908

[B42] Plewka P. Thompson A. Szymanski M. Nuc P. Knop K. Rasinska A. (2018). A stable tRNA-like molecule is generated from the long noncoding RNA *GUT15* in *Arabidopsis*. *RNA Biol.* 15 726–738. 29561243 10.1080/15476286.2018.1445404 PMC6152437

[B43] Robinson M. D. McCarthy D. J. Smyth G. K. (2010). edgeR: a Bioconductor package for differential expression analysis of digital gene expression data. *Bioinformatics* 26 139–140. 10.1093/bioinformatics/btp616 19910308 PMC2796818

[B44] Rune B. Heidi Kiil B. (2010). Overview of retinoid metabolism and function. *J. Neurobiol.* 66 606–630. 10.1002/neu.20242 16688755

[B45] Shim J. Seo H. Choi Y. Yoo I. Lee C. K. Hyun S. H. (2013). Analysis of legumain and cystatin 6 expression at the maternal-fetal interface in pigs. *Mol. Reprod. Dev.* 80 570–580. 10.1002/mrd.22192 23686917

[B46] Sigurgeirsson B. Åmark H. Jemt A. Ujvari D. Westgren M. Lundeberg J. (2017). Comprehensive RNA sequencing of healthy human endometrium at two time points of the menstrual cycle. *Biol. Reprod.* 96 24–33. 28395321 10.1095/biolreprod.116.142547

[B47] Simmons D. G. Kennedy T. G. (2002). Uterine sensitization-associated gene-1: a novel gene induced within the rat endometrium at the time of uterine receptivity/sensitization for the decidual cell reaction. *Biol. Reprod.* 67 1638–1645. 10.1095/biolreprod.102.006858 12390898

[B48] Song G. Bazer F. W. Spencer T. E. (2007). Pregnancy and interferon tau regulate RSAD2 and IFIH1 expression in the ovine uterus. *Reproduction* 133 285–295. 10.1530/rep-06-0092 17244754

[B49] Spencer T. E. Burghardt R. C. Johnson G. A. Bazer F. W. (2004a). Conceptus signals for establishment and maintenance of pregnancy. *Anim. Reprod. Sci.* 2:49. 10.1016/j.anireprosci.2004.04.014 15271478

[B50] Spencer T. E. Johnson G. A. Bazer F. W. Burghardt R. C. (2004b). Implantation mechanisms: insights from the sheep. *Reproduction* 128 657–668. 10.1530/rep.1.00398 15579583

[B51] Spencer T. E. Johnson G. A. Bazer F. W. Burghardt R. C. (2007a). Fetal-maternal interactions during the establishment of pregnancy in ruminants. *Soc. Reprod. Fertil. Suppl.* 64 379–396. 10.5661/rdr-vi-379 17491160

[B52] Spencer T. E. Johnson G. A. Bazer F. W. Burghardt R. C. Massimo P. (2007b). Pregnancy recognition and conceptus implantation in domestic ruminants: roles of progesterone, interferons and endogenous retroviruses. *Reprod. Fertil. Dev.* 19 65–78. 10.1071/rd06102 17389136

[B53] Stewart M. D. Stewart D. M. Johnson G. A. Vyhlidal C. A. Burghardt R. C. Safe S. H. (2001). Interferon-tau activates multiple signal transducer and activator of transcription proteins and has complex effects on interferon-responsive gene transcription in ovine endometrial epithelial cells. *Endocrinology* 142 98–107. 10.1210/endo.142.1.7891 11145571

[B54] Vineet Kumar M. Rajesh Kumar J. Vijay K. Anubha J. Sangappa C. Jasna Jagan M. (2013). Transforming growth factor-beta 1 (TGF-B1) liberation from its latent complex during embryo implantation and its regulation by estradiol in mouse. *Biol. Reprod.* 89:84. 10.1095/biolreprod.112.106542 23926286

[B55] Wang Y. Xue S. Liu X. Liu H. Hu T. Qiu X. (2016). Analyses of long non-coding RNA and mRNA profiling using RNA sequencing during the pre-implantation phases in pig endometrium. *Sci. Rep.* 6:20238. 10.1038/srep20238 PMC4731748 26822553

[B56] Wango E. O. Wooding F. B. P. Heap R. B. (1990). The role of trophoblast binucleate cells in implantation in the goat: a quantitative study. *Placenta* 11 381–394. 10.1016/s0143-4004(05)80214-0 2082345

[B57] Xia H.-F. Jin X.-H. Song P.-P. Cui Y. Liu C.-M. Ma X. (2010). Temporal and spatial regulation of miR-320 in the uterus during embryo implantation in the rat. *Int. J. Mol. Sci.* 11 719–730. 10.3390/ijms11020719 20386663 PMC2852863

[B58] Xu J. Bai J. Zhang X. Lv Y. Gong Y. Liu L. (2016). A comprehensive overview of lncRNA annotation resources. *Brief. Bioinform.* 18 236–249. 10.1093/bib/bbw015 26944085

[B59] Young M. D. Wakefield M. J. Smyth G. K. Oshlack A. (2010). Gene ontology analysis for RNA-seq: accounting for selection bias. *Genome Biol.* 11:R14. 10.1186/gb-2010-11-2-r14 PMC2872874 20132535

[B60] Zhan S. Dong Y. Zhao W. Guo J. Zhong T. Wang L. (2016). Genome-wide identification and characterization of long non-coding RNAs in developmental skeletal muscle of fetal goat. *BMC Genomics* 17:666. 10.1186/s12864-016-3009-3 27550073 PMC4994410

[B61] Zhang C. Gao L. Xu E. Y. (2016). LncRNA, a new component of expanding RNA-protein regulatory network important for animal sperm development. *Semin. Cell Dev. Biol.* 59 110–117. 10.1016/j.semcdb.2016.06.013 27345292

[B62] Zhang X. Xu Y. Fu L. Li D. Dai X. Liu L. (2018). Identification of mRNAs related to endometrium function regulated by lncRNA CD36–005 in rat endometrial stromal cells. *Reprod. Biol. Endocrinol.* 16:96. 10.1186/s12958-018-0412-4 PMC6190555 30322386

[B63] Złotkowska A. Andronowska A. (2019). Variable chemokine expression in porcine trophoblasts and endometrium during the peri-implantation period. *Theriogenology* 131 16–27. 10.1016/j.theriogenology.2019.03.010 30928625

